# Supplementary material for: Individual and contextual factors affect the implementation fidelity of youth-friendly services, northwest Ethiopia: A multilevel analysis
Source: PLoS One. 2022 Feb 10;17(2):e0263733. doi: 10.1371/journal.pone.0263733 (PMC8830631; doi:10.1371/journal.pone.0263733)
Supplement: Attachment — Submitted filename: Review - PONE-D-21-19080.docx [file pone.0263733.s001.docx]

Review (Manuscript: PONE-D-21-19080)

August 05, 2021

This is a good paper for the field of implementation science. Youth-friendly service programs (YFS) are a fantastic way to link and retain youth in care services. Given the broad implementation of the YFS program in parts of Ethiopia (as noted by the authors), this research is necessary to the field. Overall, the approach to assessing fidelity I also think is valuable. However, there were some points in the manuscript that confused me as a reader. To use the Carroll et al. (2007) study you cited, implementation fidelity is focused on the degree to which an intervention is delivered as intended. In my view, an evaluation of implementation will be focused on the health facility and the providers. This is not to say that clients (i.e., youth, adolescents, etc.) cannot evaluate fidelity, but that their role in measuring the success of the intervention is different.

And this is where I came off confused. You, the authors, are looking at the fidelity of implementation, and your focus appears to be clients, those youth who seek care services from the facilities in Gondar, Ethiopia. And you don’t do a good job explaining why this became your target population. Typically, the clients (i.e., youth) will be used to assess adherence to the intervention. Then, for example, a high adherence becomes an indicator of the success of implementation.

See below for more specific comments about different sections of the manuscript.

**Introduction**

Line 78, I think you meant to write fidelity of implementation and not “fidelity f implementation”

I may be misunderstanding the manuscript, but why is the question of fidelity being posed to youth? Who is the target population? Fidelity would seem to be an issue for the program implementers and providers. The providers and program implementation team would be the ones to assess whether providers delivered YFS as it was designed.

The question for the youth would be one of adherence. The success of YFS for youth would be whether they came for services and continued to come for services for the length of the program can be given a better assessment of w

**Methods**

The section on instrument development is a bit long, with considerable repetition. For example, you mention that aspects of the final instrument were adapted from the WHO standard on Line 163 and 216. Consider shortening this section, given that you are interested in one component of the instrument, which is fidelity. It would help if you also considered writing a manuscript on the instrument development or the study protocol. The development of the instrument is itself an interesting process with worthwhile contributions to the literature.

What are some examples of questions used in the fidelity component of the instrument?

It is not clear why the study (or the part of the study reported in this manuscript) has both youth and providers as the study population. Specifically, was the instrument given to providers the same as the instrument given to the youth participants? Why is that? Providers would have a different perspective on issues around fidelity; I would expect you to look to capture those differences.

And again, if it is the fidelity of implementation, why do you need the perspective of the youth. I don’t imagine that the youth, who are clients of these health care facilities, would have a significant say in how a program is implemented. Or at least they would have a lesser say compared to providers.

How was fidelity understood and defined by the authors? Reading the manuscript, I feel you assume your readers have the same understanding of fidelity as you do. For example, in the methods the items used to capture fidelity, what domains do they cover. You mention that it is based on an existing instrument used by the WHO. But what aspects/constructs of fidelity does it cover?

**Results**

You report youth and health care providers together in Table 01; should that not be separated? The factors that impact providers when it comes to the question of fidelity I imagine, are distinct.

Table 02 and Table 03 cover issues that are not discussed in the methods section. That is level of youth engagement and facility-level factors are not evident in the methods. For table 02, in particular, what is meant by the level of engagement?

Table 02 could be made more clear if you categorized the engagement levels. When I reference Table 05, it is clear that the levels of engagement form part of the independent variables in your multi-level model. So, for example, if model II was adjusted for individual-level variables, then have Table 02 show this so that your readers can better connect Table 02 and Table 05.

Consider shortening the Items/ variable names in Tables 02, 03, and 05; it makes the tables clunky and challenging to read.

Discussion

Is there a reason you reference implementation in education and not the implementation in health care to support your point here? It is not wrong, but I think your argument would be stronger if you found implementation studies in the health sector and the sexual health field specifically to support notions of fidelity of implementation (Lines 512 - 514).

So, what are the practice implications of your study findings? YFS programs are demonstrated in the literature to be effective and successful ( I think several clinical studies bear this out). The question then is the state of fidelity of implementation in Gandor, Ethiopia, and more importantly, what would the iteration and scale-up of YFS programs in Ethiopia look like, given your analysis data? I think this should be a significant focus of your discussion. Ethiopia has already begun implementing YFS programs, so what will need to change to enhance fidelity in light of the results?

**Limitation**

You may wish to say a bit more regarding the limitations of the study. What do the survey items on fidelity leave out when it comes to understanding implementation?
